# Pose2Sim: An End-to-End Workflow for 3D Markerless Sports Kinematics—Part 2: Accuracy

**DOI:** 10.3390/s22072712

**Published:** 2022-04-01

**Authors:** David Pagnon, Mathieu Domalain, Lionel Reveret

**Affiliations:** 1 Laboratoire Jean Kuntzmann, CNRS UMR 5224, Université Grenoble Alpes, 38400 Saint Martin d’Hères, France; lionel.reveret@inria.fr; 2 Institut Pprime, CNRS UPR 3346, Université de Poitiers, 86360 Chasseneuil-du-Poitou, France; mathieu.domalain@univ-poitiers.fr; 3 INRIA Grenoble Rhône-Alpes, 38330 Montbonnot-Saint-Martin, France

**Keywords:** markerless motion capture, sports performance analysis, kinematics, computer vision, OpenPose, OpenSim, deep learning, concurrent validity, accuracy

## Abstract

Two-dimensional deep-learning pose estimation algorithms can suffer from biases in joint pose localizations, which are reflected in triangulated coordinates, and then in 3D joint angle estimation. Pose2Sim, our robust markerless kinematics workflow, comes with a physically consistent OpenSim skeletal model, meant to mitigate these errors. Its accuracy was concurrently validated against a reference marker-based method. Lower-limb joint angles were estimated over three tasks (walking, running, and cycling) performed multiple times by one participant. When averaged over all joint angles, the coefficient of multiple correlation (CMC) remained above 0.9 in the sagittal plane, except for the hip in running, which suffered from a systematic 15° offset (CMC = 0.65), and for the ankle in cycling, which was partially occluded (CMC = 0.75). When averaged over all joint angles and all degrees of freedom, mean errors were 3.0°, 4.1°, and 4.0°, in walking, running, and cycling, respectively; and range of motion errors were 2.7°, 2.3°, and 4.3°, respectively. Given the magnitude of error traditionally reported in joint angles computed from a marker-based optoelectronic system, Pose2Sim is deemed accurate enough for the analysis of lower-body kinematics in walking, cycling, and running.

## 1. Introduction

As coaching athletes implies observing and understanding their movements, motion analysis is essential in sports. It helps improving movement efficiency, preventing injuries, or predicting performances. According to Atha [1], an ideal motion analysis system involves the collection of accurate information, the elimination of interference with natural movement, and the minimization of capture and analysis times. Currently, reference methods in sports analysis remain marker-based. These methods, also known as MoCap (motion capture) procedures, are mostly concerned with accuracy, despite the fact that marker placement hinders natural movement and is time consuming. Therefore, several markerless technologies are being examined to solve these issues. The main candidates are either based on Inertial Measurement Units (IMUs) [2,3], depth cameras [4,5,6], or a network of RGB cameras [7,8,9]. IMUs avoid all camera-related issues such as complex setup and calibration, potential self- and gear obstructions, and can operate in real time; however, they need to be worn by the athlete and are sensitive to drift over time, and to ferromagnetic disturbances. Depth cameras offer more information than RGB cameras but they hardly work in direct sunlight nor at a distance over 5 m [10]. On the other hand, a network of RGB cameras does not assume any particular environment, and it does not hinder the athlete’s movement and focus, but it requires delicate calibration, complex setup, large storage space, and high computational capacities. The technology, however, is still maturing and some light-weight systems such as BlazePose [11] or UULPN [12] are being proposed, which can operate in real time on a mobile phone; however, they are still not quite as accurate as required for quantitative motion analysis.

We focus on the latter approach, and more specifically on methods triangulating 2D joint center estimations from a network of several calibrated RGB cameras. The most common evaluation metric is the Mean Per Joint Position Error (MPJPE), which is the average Euclidian distance between the estimated joint coordinate and its ground truth. A large part of studies investigating 3D joint center estimation choose to triangulate the output of OpenPose [13], a deep-learning algorithm estimating 2D joint coordinates from videos. Their MPJPE usually lies between 30 and 40 mm [14,15,16]. Ankle MPJPEs are within the margin of error of marker-based technologies (1–15 mm), whereas knee and hip MPJPEs are greater (30–50 mm). These errors are systematic and likely due to “ground-truth” images being mislabeled in the training dataset [17]. Triangulation from other 2D deep-learning algorithms (such as AlphaPose [18] and DeepLabCut [19]) have also been compared [17]. AlphaPose results are similar to OpenPose’s; however, DeepLabCut errors are substantially higher.

Numerous studies have focused on the accuracy of 3D joint center estimation, but far fewer have examined 3D joint angle estimation. D’Antonio et al. computed direct flexion-extension angles for the lower limb from two cameras processed with OpenPose [20]. Range of Motion (ROM) errors lay between 2.8° and 14.1°. Wade et al. calculated frontal and sagittal knee and hip angles with OpenPose, AlphaPose, and DeepLabCut [21]. They deemed the method accurate enough for assessing step length and velocity, but not for joint angle analysis. AniPose offers a toolkit for triangulating 2D poses from DeepLabCut [22]. To our knowledge, it has only been concurrently validated for index finger angles in the sagittal plane, resulting in a root-mean-square error of 7.5 degrees [23]. Theia, a commercially available software package for markerless analysis, uses its own patent-protected 2D pose estimator and triangulation procedure, and runs a skeletal model to constrain the results to physically consistent poses and movements [24]. Their root-mean-square error (RMSE) compared to a marker-based method ranged between 2.6° and 13.2°.

We previously proposed Pose2Sim [25], an open-source markerless kinematics workflow using a network of calibrated RGB cameras, bridging OpenPose [13] to OpenSim. OpenSim is open-source 3D biomechanical analysis software that uses a multi-body optimization approach to solve inverse kinematics [26,27]. Our previous study [25] showed that Pose2Sim was robust to dark and blurry images (0.5 gamma compression and 5.5 cm Gaussian blur), to 1 cm random calibration errors, and to using as few as four cameras. Because Needham et al. showed that the quality of markerless results were task specific [28], we examined walking, running, and cycling. The objective of the present study was to concurrently evaluate Pose2Sim’s lower-limb 3D accuracy on the same tasks with a marker-based method.

## 2. Materials and Methods

### 2.1. Participant and Protocol

**Figure 1:**
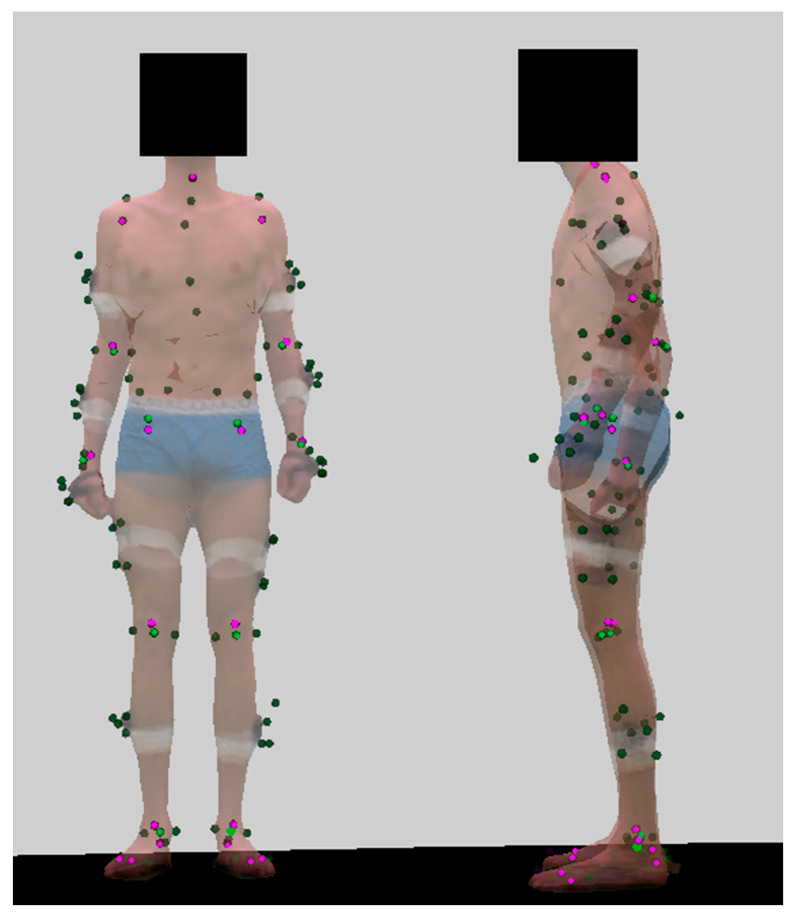
Triangulated anatomical markers and clusters (dark green), calculated joint centers (light green), and OpenPose BODY_25B keypoints (pink) on a textured mesh. OpenPose’s eyes and ears keypoints were excluded [25]. Mesh opacity was set to 0.5 in order to make all points visible. This view made it possible to precisely place OpenPose triangulated keypoints on the OpenSim model.

One adult male participant (1.89 m, 69 kg) was equipped with 83 reflective markers inspired from the CAST marker set [29], composed of 35 anatomical markers, and 12 clusters of 4 markers (Figure 1). He was asked to perform three tasks: walking, running, and cycling at a regular pace back and forth across the capture space, following a regular pulsing sound (see previous article for further details [25]). He provided his written consent prior to participating.

### 2.2. Data Collection

**Figure 2:**
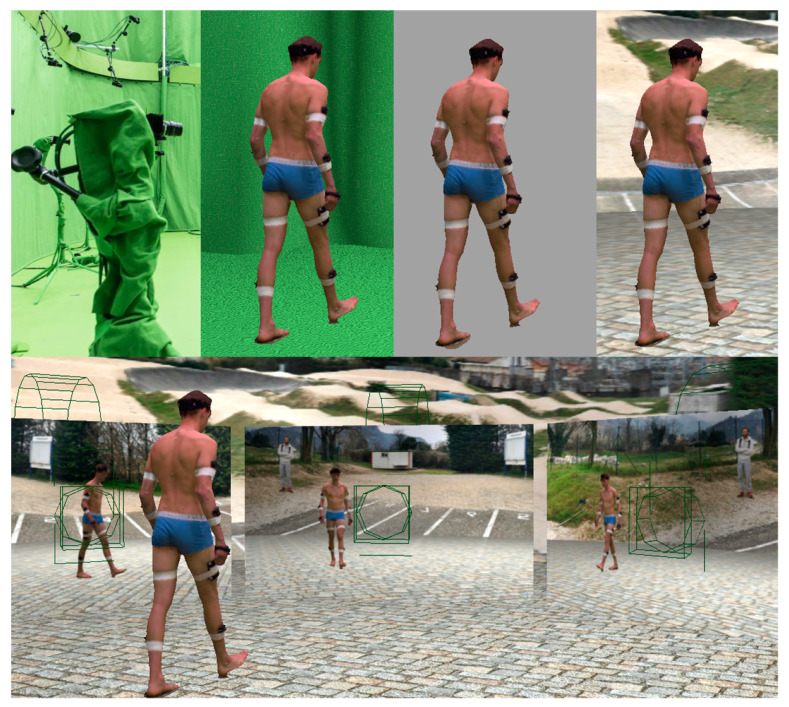
Participant’s 3D textured meshes were extracted using 68 video cameras in the studio, and then placed in a virtual environment. The scene was then filmed from 8 virtual cameras.

All tasks were performed in a room equipped with a green background for optimal segmentation of the subject with respect to the background, and 3D animated mesh extraction using a visual hull approach at each video frame [30]. Twenty opto-electronic cameras captured the 3D coordinates of the markers, and 68 video cameras allowed retrieval of 3D textured meshes of the participant, which we subsequently placed in a virtual environment and filmed from 8 virtual cameras (Figure 2). This gave us the opportunity to assess the robustness of our protocol (see Part 1 of this series of articles [25]), and for overlaying triangulated markers, calculated joint centers, and OpenPose keypoints to the extracted mesh. This was particularly useful to correctly place OpenPose keypoints on the OpenSim model, i.e., with a systematic offset as regards true joint centers [17] (Figure 1). The acquisition was restricted in terms of 3D volume covered by both systems and data storage, resulting in the analysis of 8, 13, and 13 cycles of walking, running, and cycling, respectively. Once 3D point coordinates were retrieved, both systems underwent processes that were as close to each other as possible: coordinates were sampled at 30 Hz, then they were filtered with a 4th-order 6 Hz low-pass Butterworth filter (which efficiently filtered out noise without underestimating peak values, including in extremities); heel strikes were detected in both cases with the Zeni et al. method [31]; stride duration was determined as the inverse of the frequency of the metronome followed by the participant; and inverse kinematics were optimized with the same OpenSim skeletal model.

### 2.3. Pose2Sim Kinematics

**Figure 3:**
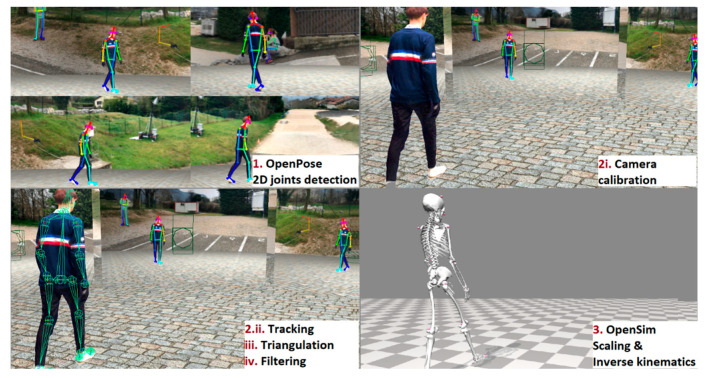
Pose2Sim full pipeline: (1) OpenPose 2D joint detection; (2i) camera calibration; (2ii–iv) tracking the person of interest, triangulating his coordinates, and filtering them; (3) constraining the 3D coordinates to a physically consistent OpenSim skeletal model.

All videos from our virtual cameras were processed by OpenPose (version 1.6), which delivered 2D joint coordinates for each view. We used the OpenPose experimental BODY_25B model (Figure 1) with the highest accuracy parameters [32]. The Pose2Sim workflow was then used to track the person of interest, robustly triangulate the OpenPose 2D joint coordinates, and filter the resulting 3D coordinates. Then this output was fed to our OpenSim setup to constrain the results to physically consistent kinematics (Figure 3; more details in our previous study [25]). The code is freely available on https://gitlab.inria.fr/perfanalytics/pose2sim (accessed on 26 March 2022).

Pose2Sim comes with a generic OpenSim skeletal model that has been slightly improved since the last study [25]. It was adapted from the human gait full-body model [33] and the lifting full-body model [34]. Although the spine of the gait model is represented as a single rigid bone, it is articulated in the lifting model, and each lumbar vertebra is constrained to the next one. This is more accurate for activities for which the spine is bent, such as cycling. However, the knee joint is more accurately defined in the gait model: abduction/adduction and internal/external rotation angles are constrained to the flexion/extension angle, whereas they are simply ignored in the lifting one. This also improves the estimation of knee flexion. All else being equal, as we want our model to be as versatile as possible, we used the spine definition of the lifting model, and the knee definition of the gait model. Since we did not investigate muscle-related issues, they were removed to decrease computation time. Since no keypoint would have accounted for it, wrist flexion and deviation were locked at 0°, and arm pronation/supination was locked at 90°. Conversely, the translation of the pelvis was unlocked, in addition to the subtalar angle; and hip flexion was limited to 150° instead of 120° (which was not enough for the pedaling task). With regards to our previous study [25], marker placement was also improved in the OpenSim model. The average systematic offset between OpenPose-triangulated keypoints and MoCap-calculated joint centers [17] was measured on our 3D overlay view (Figure 1), and was taken into account when manually placing OpenPose keypoints onto the OpenSim unscaled model.

OpenSim (version 4.2) was used to scale the model to the participant on a T-pose, and then inverse kinematics was performed. Scale factors were computed with measurement-based scaling, i.e., by computing the ratio of distances between keypoints on the model, and experimental keypoints provided by the coordinates file of triangulated OpenPose data. Static pose weights were all set to 1, apart from Nose and Head keypoints which were set to 0.1, and Shoulder and Hip keypoints were set to 2. The participant was standing upright with feet flat during his T-pose, so we set a weight of 1 for a zero angle in pelvis list, pelvis tilt, L5-S1 flexion, and ankle angles. The offset in machine-learning-based joint center estimations has been demonstrated to be systematic and not dependent on the subject [17] (nor on the operator); hence, once this bias has been taken into account in the generic model, the markers’ adjustment step is unnecessary. Keypoint weight markers for inverse kinematics were the same as for scaling.

### 2.4. Marker-Based Kinematics

The captured markers were automatically identified with an AIM procedure within the Qualisys Track Manager software (version 2019.1). Joint centers were then calculated. The centers of ankles, knees, wrists, and elbows were defined as the midpoints between the malleoli/epicondyles/styloids since it has been shown that when executed on a lean participant, functional methods do not improve the reliability of the kinematics of running [35]. Hip joint center was defined with a functional method [36]. The OpenSim model used for marker-based scaling and inverse kinematics was the same as the Pose2Sim model. Scale factors were computed in a similar way, but with marker data rather than with OpenPose keypoints. Weights proposed by the inverse kinematics solver of OpenSim were set to 5 for joint centers, to 1 for cluster markers, and to 2 for other anatomical markers. Inverse kinematics was processed with the same marker weights.

### 2.5. Statistical Analysis

**Figure A1:**
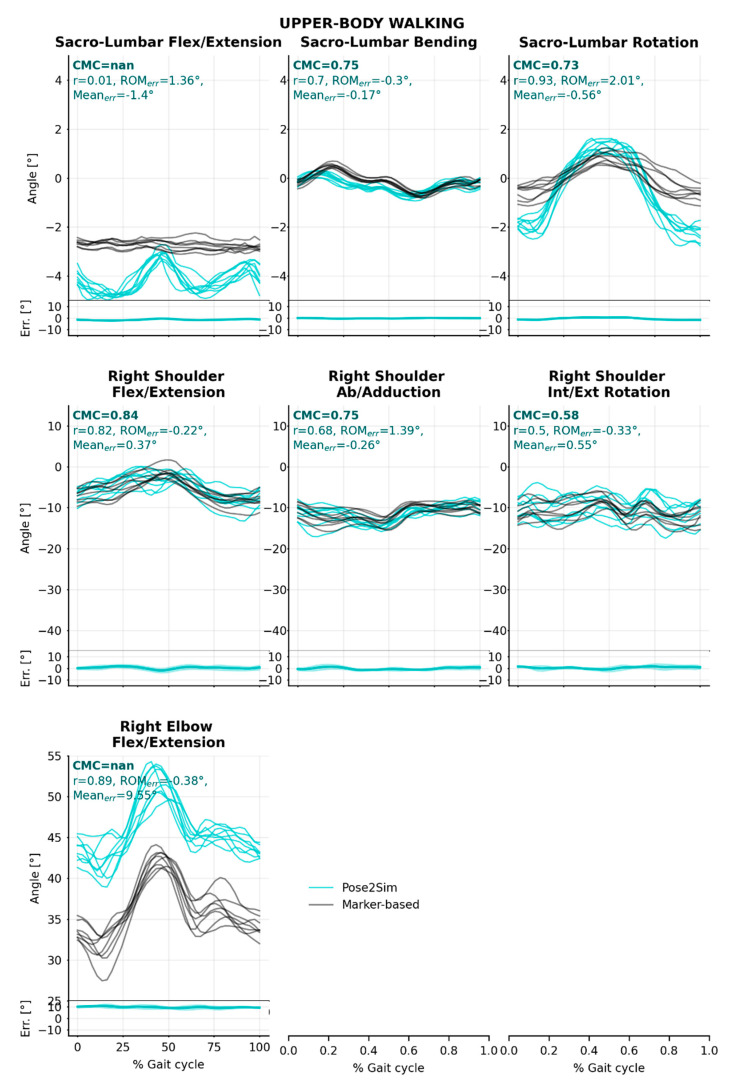
Pose2Sim (cyan) and marker-based (black) sacro-lumbar and upper-body joint angles for the walking task. Coefficient of multiple correlation (CMC) is indicated, and broken down into, respectively, Pearson’s coefficient (r) for correlation assessment, range of motion errors (ROM_err_) for gain, and overall mean error (Mean_err_) for offset. Mean error and standard deviations are also represented at the bottom of the graphics.

**Figure A2:**
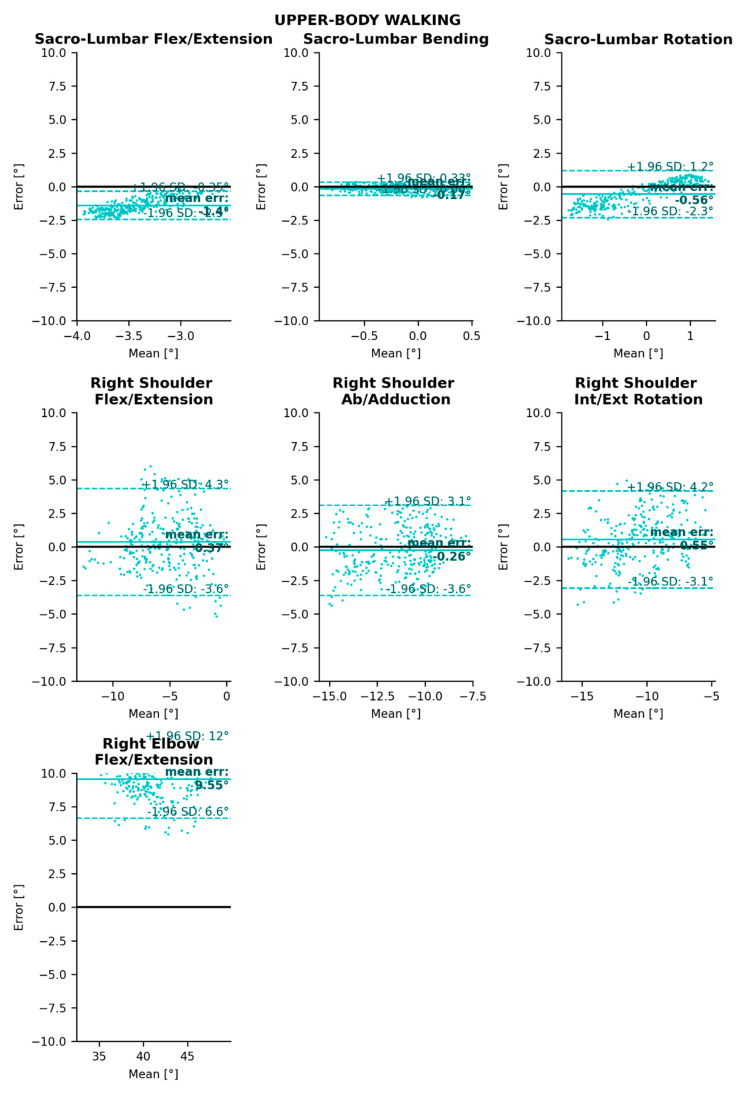
Bland–Altman analysis of sacro-lumbar and upper-body joint angle errors for the walking task. Mean bias is represented as a horizontal solid, bold line, and 95% limits of agreement are represented as dotted lines.

Since the participant did not report any locomotion impairment and the captured movements were mostly symmetrical, we only analyzed the right side. Our study focuses on the lower limb, but results for upper limb and sacro-lumbar joints are detailed in the Appendix A (Figure A1, Figure A2, Figure A5, Figure A6, Figure A9 and Figure A10) for information. The analyzed angles were ankle flexion/extension, subtalar angle, knee flexion/extension, and hip flexion/extension, abduction/adduction, and internal/external rotation provided by the OpenSim inverse kinematics procedure.

First, Pose2Sim scale factors were compared to marker-based ones, and RMS errors were reported and compared to OpenSim’s best practice rules. Then, the overall similarity of paired angle waveforms was assessed with a special formulation of the coefficient of multiple correlation (CMC), specifically designed to compare different protocols or measurement systems [37]. The CMC gives a single result taking into account differences in correlation, gain, and offset. It reaches 1 if the curves are perfectly overlapped, and drops to zero if the curves are very dissimilar, or even to complex values (reported as “nan” hereafter). This is, for example, the case if the mean inter-protocol offset (averaged over time and trials) exceeds the grand mean ROM, which results in taking the square root of a negative number. CMC values are deemed good if between 0.75 and 0.84, very good if between 0.85 and 0.94, and excellent if above 0.95 [37]. The CMC results were then broken down to take an in-depth look into correlation, gain, and offset, separately. The strength of the linear relationship between kinematic analysis systems was assessed with the Pearson’s r correlation coefficient. Gain was evaluated by computing the paired ROM differences. Once normality of the ROM errors was checked with a Shapiro–Wilk test [38], we computed related t-tests to verify whether the error was significant. Mean inter-protocol offset angle, hereafter called mean error, was one of the outputs of the subsequent Bland–Altman analysis [39,40]. Once normality of the paired means of the angle differences was verified, we determined if the mean markerless angles were significantly different to the mean marker-based ones.

The Bland–Altman analysis gives some more information about the agreement between the considered markerless and marker-based systems [39,40]. It consists of plotting the difference between the values given by both systems against their mean, for all angular points at all time instances. Limits of agreement were defined as the interval within which 95% of data will be found, i.e., between mean difference ±1.96 standard deviation, provided that the differences follow a normal distribution. Bland–Altman plots also help to identify the potential presence of heteroscedasticity, i.e., the fact that the spread of the error may depend on the angle magnitude [40].

Finally, root-mean-square errors (RMSE), mean errors (Mean_err_), and ROM errors (ROM_err_) were computed for the walking task to enable comparison with previously published metrics obtained using Theia3D, a commercial markerless solution [24], and Xsens [41], a commercial system based on IMUs. Theia3D’s ROM results were approximated from reported graphics along the flexion/extension degree of freedom.

## 3. Results

### 3.1. Inverse Kinematics: CMC, Correlation, Gain, Offset

Inverse kinematics is successful when OpenSim’s global optimizer keeps the model markers close to experimental markers, i.e., when RMSE is less than 2–4 cm according to OpenSim’s best practices. This was the case for both systems (Table 1). However, RMSE was particularly higher in cycling than in walking or running.

**Figure 4:**
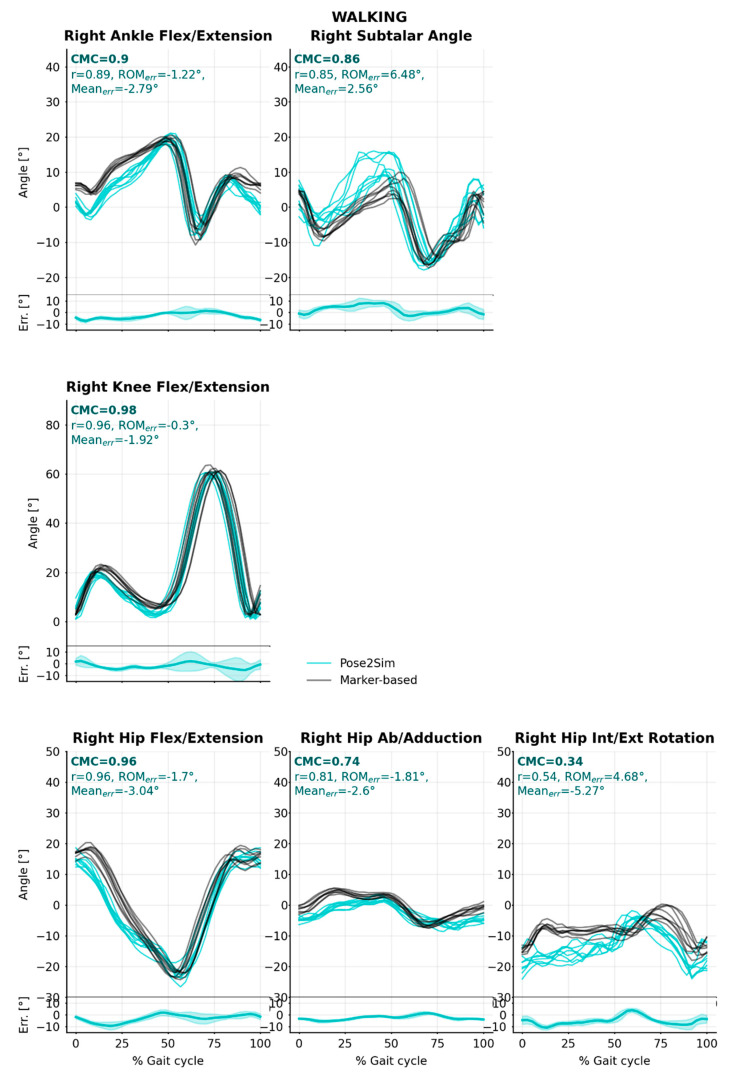
Pose2Sim (cyan) and marker-based (black) lower-body joint angles for the walking task. Coefficient of multiple correlation (CMC) is indicated, and broken down into, respectively, Pearson’s coefficient (r) for correlation assessment, range of motion errors (ROM_err_) for gain, and overall mean errors (Mean_err_) for offset. Mean error and standard deviations are also represented at the bottom of the graphics. See Appendix A for running and cycling results, and for sacro-lumbar and upper-body results.

**Figure A3:**
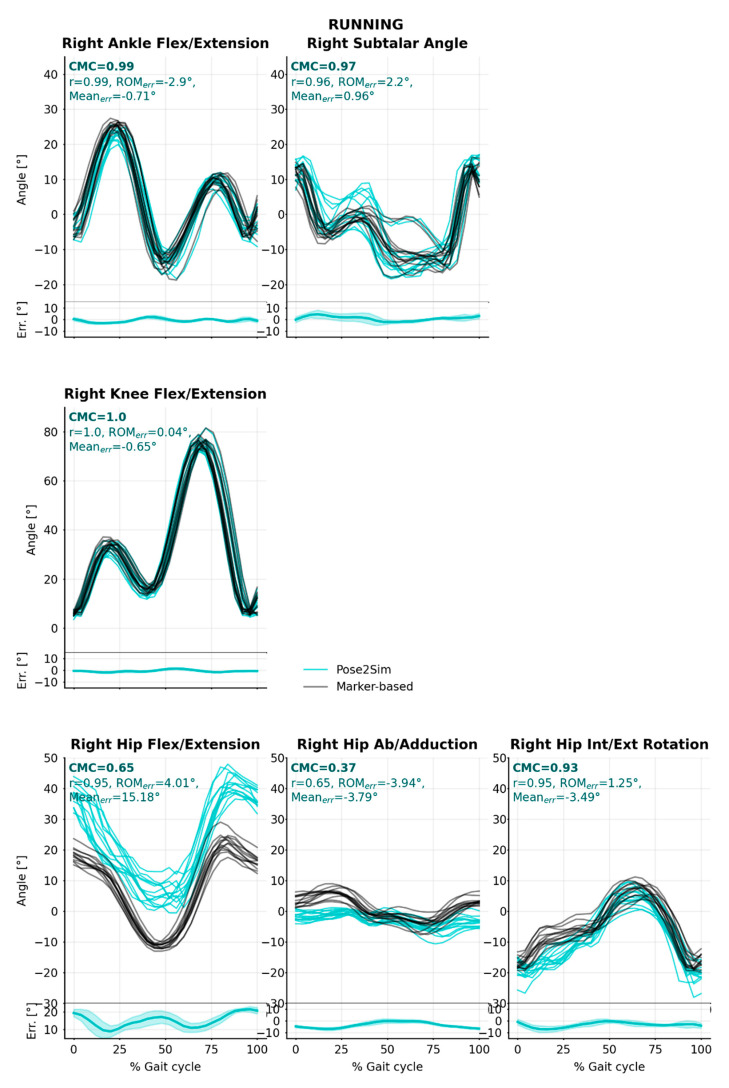
Pose2Sim (cyan) and marker-based (black) lower-body joint angles for the running task. Coefficient of multiple correlation (CMC) is indicated, and broken down into, respectively, Pearson’s coefficient (r) for correlation assessment, range of motion errors (ROM_err_) for gain, and overall mean errors (Mean_err_) for offset. Mean error and standard deviations are also represented at the bottom of the graphics.

CMC assesses waveform similarities between Pose2Sim and the marker-based reference method, by jointly evaluating correlation, gain, and offset. It was mostly very good (CMC > 0.85) to excellent (CMC > 0.95) in all tasks, all degrees of freedom, and all lower-body joints (Table 2, Figure 4, Figure A3 and Figure A7). This was especially the case along the flexion/extension degree of freedom, except for angles of the hip in running and of the ankle in cycling, for which CMC results suffered from an offset compared to the marker-based method. Hip abduction/adduction and internal/external rotation waveforms were not in good agreement (CMC < 0.75), except for the hip internal/external rotation angles in running. In cycling, all non-sagittal angles had complex CMCs, which means that no agreement was found at all.

Pearson’s r correlation coefficient results were close to the CMC ones, albeit they became very good to excellent in the two angles that were affected by an offset. When averaged over all joint angles, errors in the range of motion (ROM_err_) were 2.7° (sd = 2.1°), 2.3° (sd = 1.1°), and 4.3° (sd = 2.5°) in walking, running, and cycling, respectively. Along the flexion/extension degree of freedom, they were below 2°, 4°, and 6°, in walking, running, and cycling, respectively. Along the internal/external rotation degree of freedom, they stayed below 5°; however, they reached up to 10° along the abduction/adduction degree of freedom. Average mean angle errors (Mean_err_) were 3.0° (sd = 1.0°), 4.1° (sd = 1.6°), and 4.0° (sd = 0.59°), in walking, running, and cycling, respectively. In walking and running, mean errors remained under 5.3° in all degrees of freedom, apart from the hip flexion/extension angle in running. which was offset by 15°. Although they were noticeably larger, mean errors were always under 7° in cycling (Table 2 and Table 4, Figure A3 and Figure A7). 

**Figure 5:**
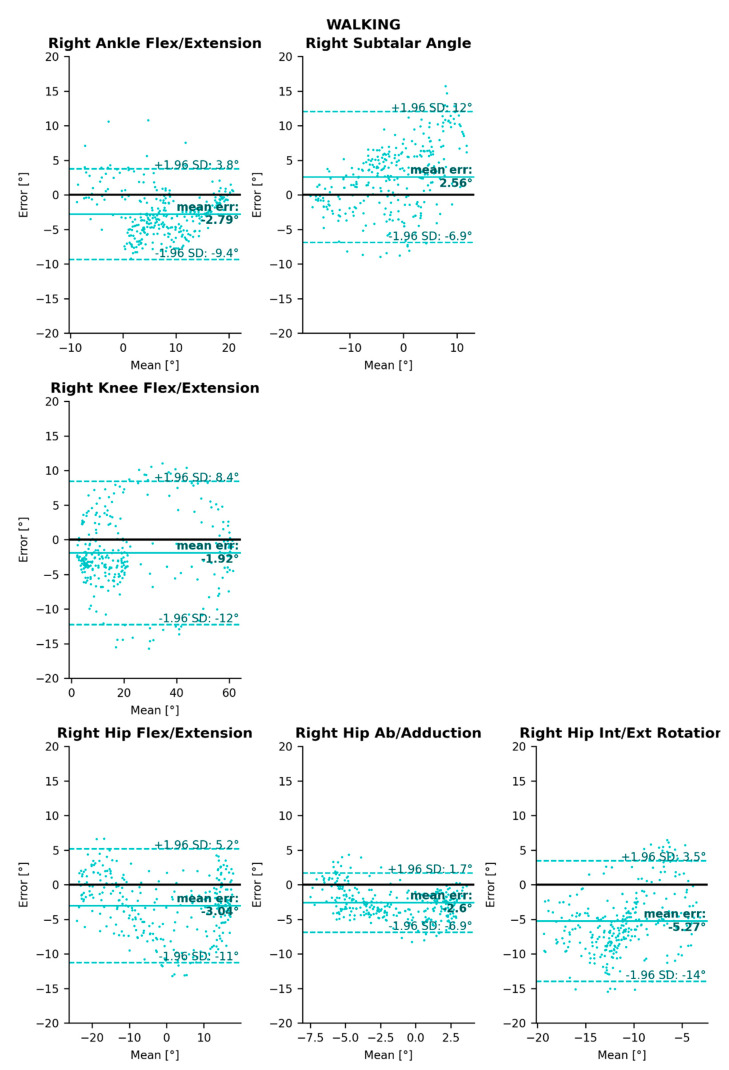
Bland–Altman analysis of lower-body joint angle errors for the walking task. Mean bias is represented as a horizontal solid, bold line, and 95% limits of agreement are represented as dotted lines. See Appendix A for running and cycling results, and for sacro-lumbar and upper-body results.

**Figure A4:**
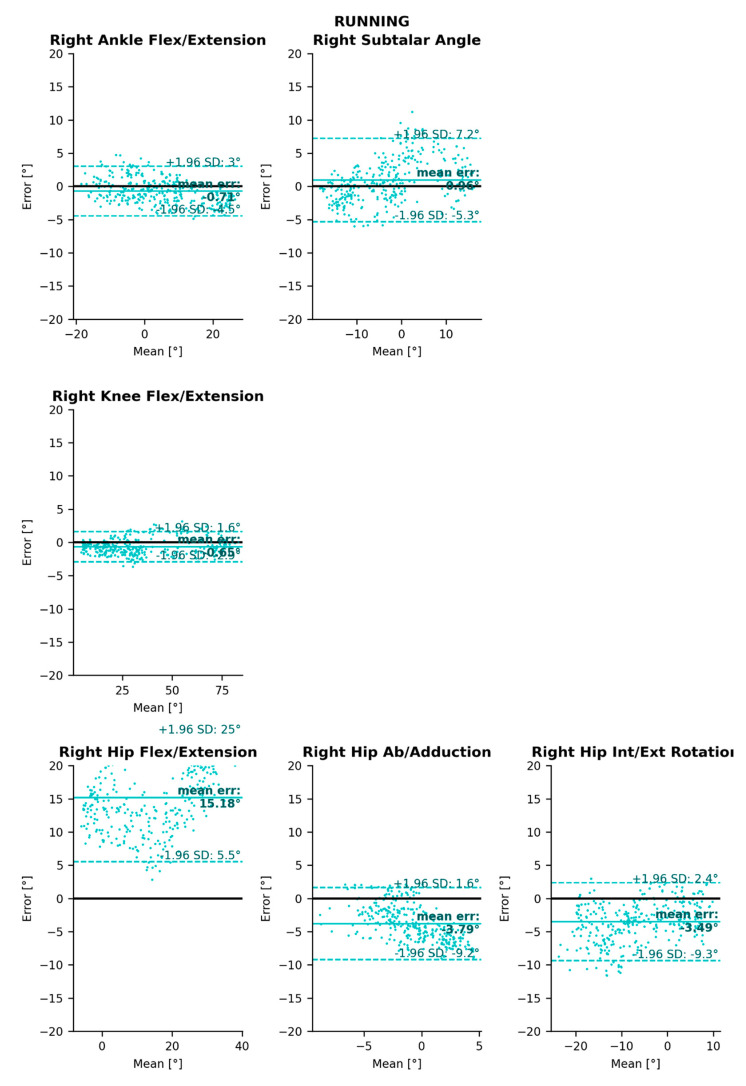
Bland–Altman analysis of lower-body joint angle errors for the running task. Mean bias is represented as a horizontal solid, bold line, and 95% limits of agreement are represented as dotted lines.

**Figure A5:**
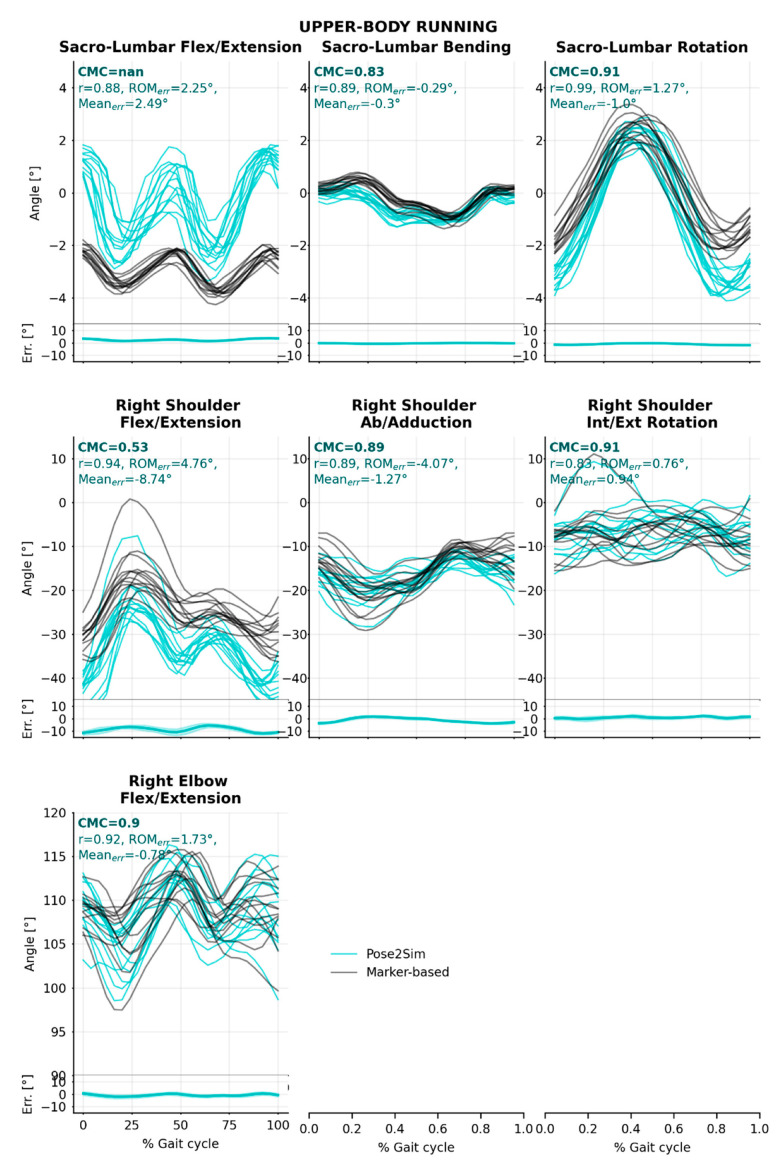
Pose2Sim (cyan) and marker-based (black) sacro-lumbar and upper-body joint angles for the running task. Coefficient of multiple correlation (CMC) is indicated, and broken down into, respectively, Pearson’s coefficient (r) for correlation assessment, range of motion errors (ROM_err_) for gain, and overall mean error (Mean_err_) for offset. Mean error and standard deviations are also represented at the bottom of the graphics.

**Figure A6:**
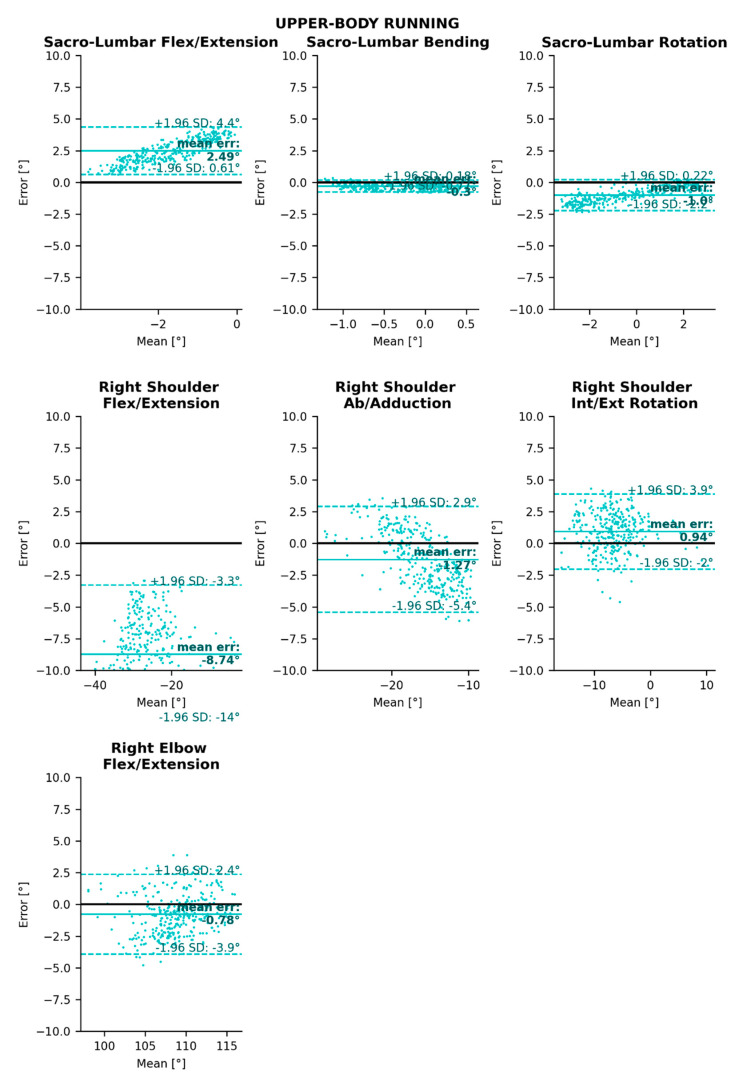
Bland–Altman analysis of sacro-lumbar and upper-body joint angle errors for the running task. Mean bias is represented as a horizontal solid, bold line, and 95% limits of agreement are represented as dotted lines.

**Figure A7:**
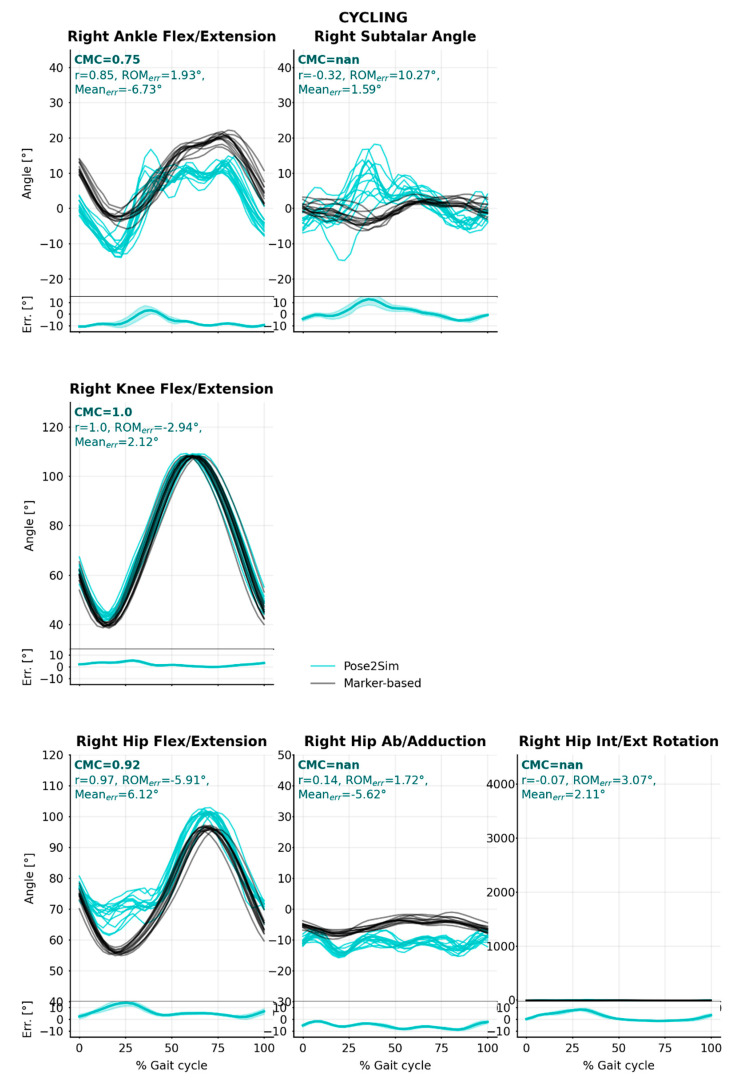
Pose2Sim (cyan) and marker-based (black) lower-body joint angles for the cycling task. Coefficient of multiple correlation (CMC) is indicated, and broken down into, respectively, Pearson’s coefficient (r) for correlation assessment, range of motion errors (ROM_err_) for gain, and overall mean errors (Mean_err_) for offset. Mean error and standard deviations are also represented at the bottom of the graphics.

**Figure A8:**
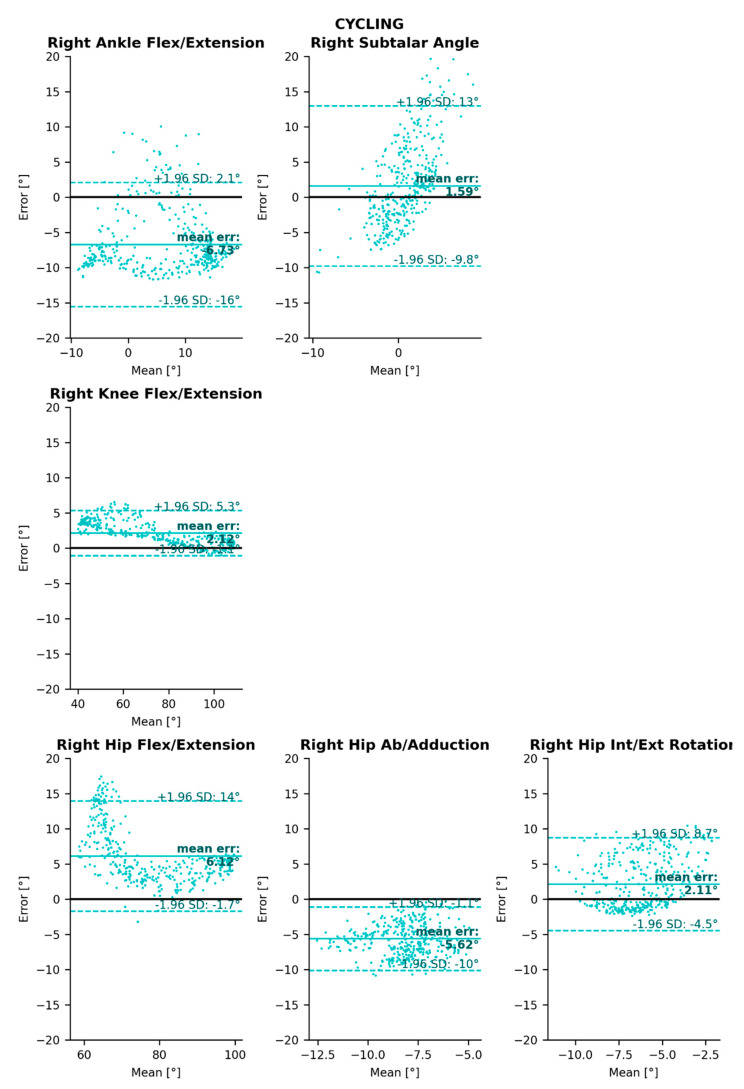
Bland–Altman analysis of lower-body joint angle errors for the cycling task. Mean bias is represented as a horizontal solid, bold line, and 95% limits of agreement are represented as dotted lines.

**Figure A9:**
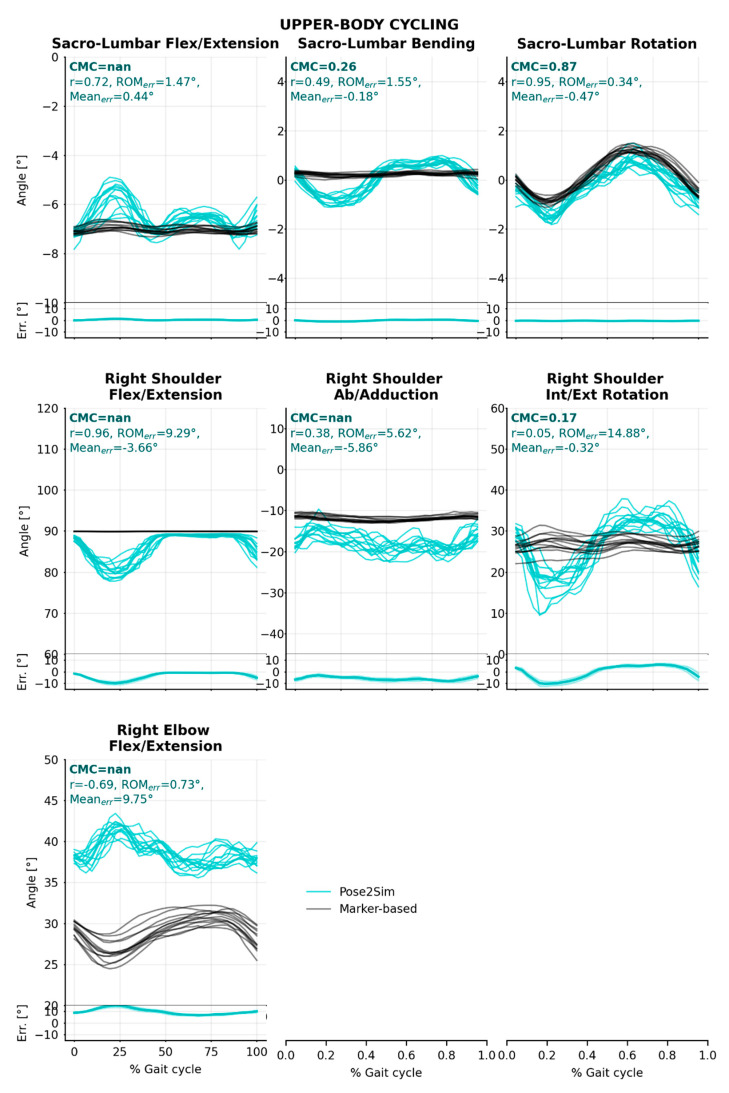
Pose2Sim (cyan) and marker-based (black) sacro-lumbar and upper-body joint angles for the cycling task. Coefficient of multiple correlation (CMC) is indicated, and broken down into, respectively, Pearson’s coefficient (r) for correlation assessment, range of motion errors (ROM_err_) for gain, and overall mean errors (Mean_err_) for offset. Mean error and standard deviations are also represented at the bottom of the graphics.

**Figure A10:**
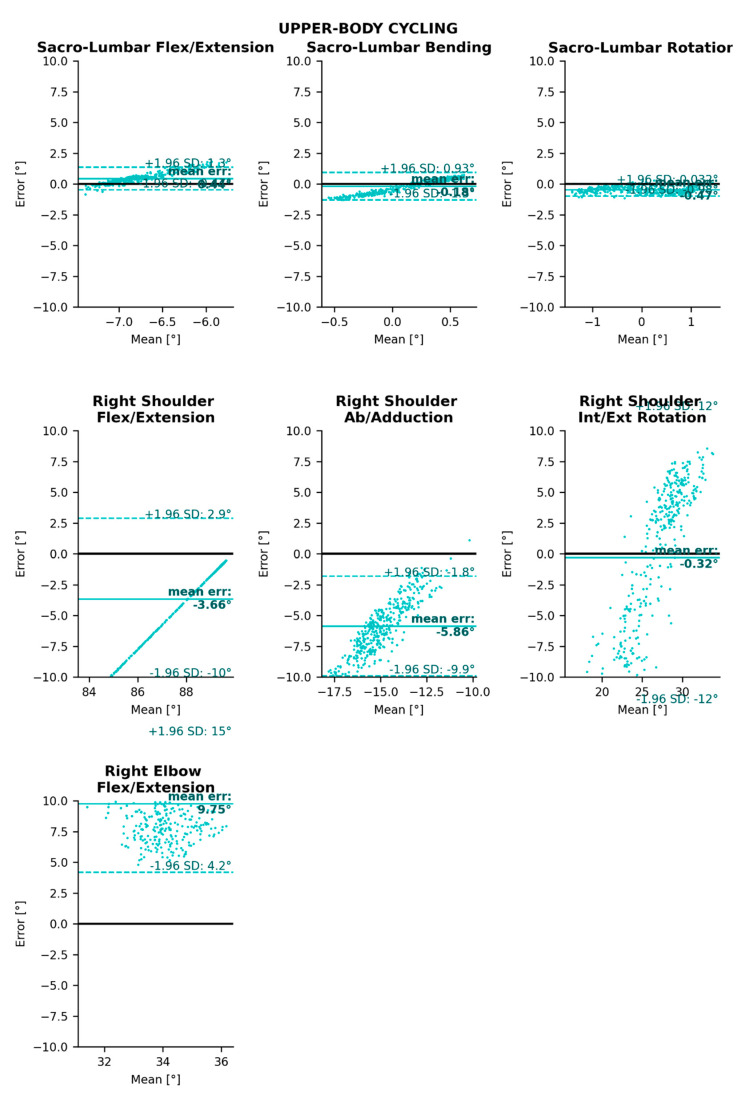
Bland–Altman analysis of sacro-lumbar and upper-body joint angle errors for the cycling task. Mean bias is represented as a horizontal solid, bold line, and 95% limits of agreement are represented as dotted lines.

Limits of Agreement (LoA) values were relatively evenly and randomly distributed among all tasks, degrees of freedom, and joints, averaging to an interval of 15° within which 95% of the errors would lie (Table 3, Figure 5, Figure A4 and Figure A8). Due to the limited range of motion of sacro-lumbar and upper-body angles, limits of agreement were smaller in these joint angles (Figure A2, Figure A6 and Figure A10). Angle magnitude did not have an influence on the spread of errors (hence the data are homoscedastic), except for the cycling task for ankle angles and flexion/extension hip angles.

### 3.2. Comparison with Other Systems

The RMSE reported by Theia3D [24] was, on average, 1.5° higher than that of Pose2Sim, and its ROM errors were consistently higher, at least along the flexion/extension degree of freedom. However, Xsens reported mean errors 0.3° lower on average, and ROM errors 1.0° lower on average (Table 4).

## 4. Discussion

### 4.1. Strengths of Pose2Sim and of Markerless Kinematics

Pose2Sim offers a way to perform a markerless kinematic analysis from multiple calibrated views, taking OpenPose results as inputs, and giving biomechanically oriented results via OpenSim. Both OpenPose and OpenSim are open-source and among the most widespread and renowned tools in their respective fields. We compared Pose2Sim lower-body results to those of a reference marker-based method, over three tasks performed by one participant: walking, running, and cycling. Both protocols were as similar as possible, and used the same constrained skeletal model in order to ensure that there was no discrepancy in results caused by different definitions of anatomical frames [42]. Pose2Sim kinematic waveforms were very similar to marker-based ones, especially in the sagittal plane. One exception to this observation was the hip angle in running, which suffered from a 15° offset due to the dearth of keypoints in this area. This led the optimization procedure to admit two solutions for the spine curvature, both mathematically and kinematically correct: one with a lordotic posture, and the other with a kyphotic posture. There was also less agreement for ankle angles in cycling, most likely because for both Pose2Sim and marker-based kinematics, keypoint/marker detections suffered from occlusions from the bike. This is corroborated by the higher RMSE between experimental and theoretical markers observed in cycling (Table 1). The similarity of waveforms among both protocols was assessed with the coefficient of multiple correlation (CMC) [37], which takes into account the concurrent effects of correlation, gain, and offset. When averaged over all lower-limb joints and all degrees of freedom, mean errors amounted to 3.0°, 4.1°, and 4.1° in walking, running, and cycling, respectively, and range of motion errors were equal to 2°, 2.3°, and 4.3°. It should be noted that, unlike ours, Theia3D [24] and Xsens [41] studies to which we compared our results involved several subjects (30 and 10, respectively.) Our study recorded with eight virtual cameras, 1 MP definition, 30 Hz framerate, and perfect calibration, whereas the Theia system recorded with eight cameras, 3 MP definition, 85 Hz, with a marker-based calibration. Hence, the comparison between accuracies of Theia3D, Xsens and Pose2Sim are given for an overview of their order of magnitude, not as a claim for exact comparison. This study focused on lower-body kinematics, although we report upper-body and sacro-lumbar kinematics in annexes for reference. It may be noted that differences between the two approaches were higher than for the lower body, and especially for the sacro-lumbar flexion.

This shows that a carefully designed skeletal model, when correctly scaled and constrained, can lead to accurate results from a markerless approach, despite poorly labeled joint centers [17,21] and despite a low number of detected keypoints. Indeed, it has been shown that the triangulation of deep-learning-based pose estimation methods produces systematic errors up to 50 mm in 3D knee and hip joint center coordinates [17]. Without the use of a skeletal model, flexion/extension lower-body angle errors in cycling have been demonstrated to be as large as 3–12° [43]. Moreover, Pose2Sim still gave relevant results when using the coordinates of only 21 triangulated keypoints coordinates (after exclusion of eye and ear keypoints). This is in line with conclusions that were previously made for marker-based approaches, implying that constrained kinematic models are resilient to marker placement and quantity [44].

The setup of Pose2Sim can be installed anywhere, i.e., directly on-site rather than in a laboratory setting. No particular attention has to be devoted to the background color, to the participant’s clothing, nor to the luminance of the recording area. No apparatus interferes with the athlete’s movement, who can fully concentrate on their performance. This is of crucial importance in the context of sports analysis. Results are not operator or subject dependent, which makes labeling, scaling, and inverse kinematics both easy and robust. It is to be noted, however, that it does not leave room for adjustment if it is needed to better monitor a specific body part. However, the operator or scientist has access to fine control on most parameters at each step of the analysis: the deep-learning 2D pose estimation model can be changed; tracking, triangulation, and filtering parameters can be adjusted; and the OpenSim model, scaling, and inverse kinematics can be entirely controlled.

### 4.2. Limits and Perspectives

Our study still has potential limitations. First, it was conducted on a limited amount of data: only 8–13 cycles per task were captured, performed by one participant, and captured at 30 Hz. Given the relatively slow and steady movements we analyzed, we believe that this framerate did not impact our results, although both marker-based and markerless kinematics would beneficiate from a higher sample frequency on more demanding activities. Note that Pose2Sim can operate at any framerate, and this limitation is only due to the settings of the video acquisition system. Although results cannot be overly generalized to other sports movements, we assume that conclusions would hold for other healthy subjects, first because the OpenPose training was done on numerous participants having different gender, race, body shape, and outfit [45]; second, because deep-learning-based pose estimation algorithms are not subject to inter-operator errors or to soft-tissue artifacts; and, third, because the OpenSim kinematic model is scaled to the participant’s anthropometry. Nonetheless, it would be worth assessing its accuracy on more challenging sports and with multiple subjects. Moreover, we used perfect virtual cameras instead of real ones. Real cameras could have induced errors due to motion blur, large distortions, or calibration errors. Our previous study, however, showed that the system was very robust to these issues, including with as little as four cameras, at least with movements such as walking, running, and cycling on an ergometer [25]. It may be interesting to try Pose2Sim with light and versatile action cameras such as GoPros, calibrated with a checkerboard. The accuracy of these cameras has already been explored on marker-based data. Although the maximum point coordinate error was about 10 times as large as that with a motion capture system (2.47 versus 0.21 mm), knee joint angles were highly correlated (joint coordinates error below 2.5°) [46].

OpenPose keypoint localization suffers from systematic offsets when compared to actual joint center positions [17]. This has been taken into account on a static pose in the OpenSim unscaled model, by shifting OpenPose keypoint placements with regard to marker-based joint centers. This was done manually, but precisely, due to our overlayed view (Figure 1). The OpenSim model was then scaled to the participant’s anatomy without the use of any MoCap procedure. However, OpenPose’s offset may not be the same when a limb is extended as when it is bent, which may influence kinematic results on extreme poses. Hence, using a pose estimation model free from systematic biases on all ranges of motion would improve kinematic accuracy, even if applying a constrained skeletal model already largely reduces the detrimental impact of low-quality 2D joint center estimations. Pose2Sim could operate with such a 2D pose estimation model, although new keypoints should then be placed afresh on the unscaled OpenSim model. Note that the training dataset of this more accurate pose estimation model should not base its labeling on markers, which could be interpreted as visual cues, which would not be available in real sports situations. However, this condition is not sufficient: the dataset should be large enough, represent a wide variety of body types and movements [47], and include images with motion blur such as found in sports videos. It is also possible to enhance the OpenPose dataset, by training it on specific sports poses, or by augmenting it with larger rotations, so that the model recognizes upside-down poses. One risk of this approach is that the model may perform better on specific extreme poses, but worse on standard ones [48]. Furthermore, detecting more keypoints would also improve results, provided that they are reliably labeled: first, it would help solve indeterminations in non-sagittal planes and in pelvis angles, without having to add constraints to the skeletal OpenSim model; then, it would allow for the analysis of more angles, especially in the pelvis, the spine, and the upper body. Finally, instead of constraining pose estimation results with a physically consistent skeletal model, it would be interesting to develop a physics-informed pose estimation model [49], which would offer the possibility of embedding the kinematics priors as early as possible in the learning process.

Currently, Pose2Sim does not work in real time, which could be interesting for sports action live analysis. Moreover, it only automatically tracks one person of interest. It would be useful to expand it to multi-person motion analysis, especially in the context of races, team sports, or combat sports. It can also be of considerable interest to train a single neural network able to detect both the human 2D pose and sports gear, such as a ball [50], skis [51], or bike parts in the context of cycling. This would help to analyze game dynamics, and to quantify posture cues related to a specific sports discipline. Other minor adjustments could be made in order to improve the triangulation and the filtering steps. Implementing Random Simple Consensus (RANSAC) triangulation [52] as an alternative to our weighted Direct Linear Transform (DLT) [25], and opting for optimal fixed-interval Kalman smoothing instead of low-pass filtering [28,53], may reduce errors, especially in large outliers.

## 5. Conclusions

Pose2Sim can use any 2D pose estimation algorithm, triangulate 2D coordinates, and constrain the resulting 3D coordinates to a physically consistent skeletal model. Desmarais et al. proposed a taxonomy of 3D pose estimation algorithms based on accuracy, robustness, and speed [54]. Accuracy was assessed in this study, and robustness was investigated in our previous study [25]; however, speed has not yet been tested. The bottleneck for computational costs, here, is by far the pose estimation system, but some neural networks are tackling this issue [11,12].

Deep-learning-based human pose estimation is making considerable and consistent progress. It is becoming more accurate, more robust, faster, and simpler to use, approaching Atha’s 1984 [1] definition of an ideal motion analysis system. Pose2Sim takes advantage of these advances, and mitigates the remaining errors by constraining these outputs to obtain physically consistent kinematics.

## Figures and Tables

**Table 1:** RMSE between experimental and theoretical markers during OpenSim inverse kinematics, for both marker-based and Pose2Sim models.

Task	Marker-Based RMSE (cm)	Pose2Sim RMSE (cm)
Walking	1.1–1.2	1.4–1.9
Running	1.5–1.7	1.5–2.1
Cycling	3.5–3.5	3.0–3.6

**Table 2:** Summary of comparisons between Pose2Sim and marker-based angle waveforms. A specific formulation of the coefficient of multiple correlation (CMC) was used, specifically designed to compare different protocols or measurement systems [37]. CMC jointly evaluates correlation, gain, and offset, which were respectively assessed with Pearson’s r coefficient, range of motion errors (ROM_err_), and mean errors (Mean_err_). * Significant at 5% level. ^1^ Although ankle subtalar angle combines abduction/adduction and internal/external rotation, it is hereafter reported in the abduction/adduction column.

		Flexion/Extension				Abduction/Adduction ^1^					Internal/External Rotation		
		CMC	R	ROM_err_	Mean_err_	CMC	r	ROM_err_	Mean_err_	CMC	r	ROM_err_	Mean_err_
Walking	Ankle	0.90	0.89	−1.22	−2.79 *	0.86	0.85	6.48 *	2.56 *	-	-	-	-
	Knee	0.98	0.96	−0.30	−1.92 *	-	-	-	-	-	-	-	-
	Hip	0.96	0.96	−1.70	−3.04 *	0.74	0.81	−1.81 *	−2.6 *	0.34	0.54	4.68 *	−5.27 *
Running	Ankle	0.99	0.99	−2.9 *	−0.71 *	0.97	0.96	2.20	0.96	-	-	-	-
	Knee	1.00	1.00	0.04	−0.65 *	-	-	-	-	-	-	-	-
	Hip	0.65	0.95	4.01 *	15.18 *	0.37	0.65	−3.94 *	−3.76 *	0.93	0.95	1.25	−3.49 *
Cycling	Ankle	0.75	0.85	1.93 *	−6.73 *	nan	−0.32	10.27 *	1.59 *	-	-	-	-
	Knee	1.00	1.00	−2.94 *	2.12 *	-	-	-	-	-	-	-	-
	Hip	0.92	0.97	−5.91 *	6.12 *	nan	0.14	1.72 *	−5.62 *	nan	−0.07	3.07 *	2.11 *

**Table 3:** Bland–Altman analysis results of 3D angle errors between Pose2Sim analysis and the reference marker-based one. Mean errors (Mean_err_) and 95% limits of agreement (LoA) are represented. * Although ankle subtalar angle combines abduction/adduction and internal/external rotation, it is hereafter reported in the abduction/adduction column.

		Flexion/Extension		Abduction/Adduction *		Internal/External Rotation	
		Mean_err_ (°)	95% LoA (°)	Mean_err_ (°)	95% LoA (°)	Mean_err_ (°)	95% LoA (°)
Walking	Ankle	−2.79	[−9.4, 3.8]	2.56	[−6.9, 12]	-	-
	Knee	−1.92	[−12, 8.4]	-	-	-	-
	Hip	−3.04	[−11, 5.2]	−2.6	[−6.9, 1.7]	−5.27	[−14, 3.5]
Running	Ankle	−0.71	[−4.5, 3.0]	0.96	[−5.3, 7.2]	-	-
	Knee	−0.65	[−2.9, 1.6]	-	-	-	-
	Hip	15.18	[5.5, 25]	−3.76	[−9.2, 1.6]	−3.49	[−9.3, 2.4]
Cycling	Ankle	−6.73	[−16, 2.1]	1.59	[−9.8, 13]	-	-
	Knee	2.12	[−1.1, 5.3]	-	-	-	-
	Hip	6.12	[−1.7, 14]	−5.62	[−10, −1.1]	2.11	[−4.5, 8.7]

**Table 4:** Pose2Sim results compared to Theia3D [24] and to Xsens [41] in the walking task. Root-mean-square error (RMSE), mean error (Mean_err_), and range of motion (ROM) are examined. Theia3D’s ROM results were approximated from reported graphics along the flexion/extension degree of freedom. Both studies to which we compared our results involved a different setup (participants, cameras, protocol, etc.), therefore the differences cannot be totally attributed to the different technologies (markerless or IMU) nor to the different algorithm (Pose2Sim or Theia3D). * Although ankle subtalar angle combines abduction/adduction and internal/external rotation, it is hereafter reported in the abduction/adduction column.

		Flexion/Extension			Abduction/Adduction *			Internal/External Rotation		
		Pose2Sim	Theia3D [24]	Xsens [41]	Pose2Sim	Theia3D [24]	Xsens [41]	Pose2Sim	Theia3D [24]	Xsens [41]
RMSE (°)	Ankle	4	6.7	-	5.1	8	-	-	-	-
	Knee	5.1	3.3	-	-	-	-	-	-	-
	Hip	5.6	11	-	3.1	2.6	-	6.6	6.9	-
Mean_err_ (°)	Ankle	2.79	-	2.15	2.56	-	1.81	-	-	-
	Knee	1.92	-	1.87	-	-	-	-	-	-
	Hip	3.04	-	2.47	2.60	-	4.83	5.27	-	3.02
ROM_err_ (°)	Ankle	−1.22	≈−10	0.40	6.48	-	1.38	-	-	-
	Knee	−0.30	≈−1	0.80	-	-	-	-	-	-
	Hip	−1.70	≈−10	2.42	−1.81	-	5.37	4.68	-	0.04

## Data Availability

The code is freely available on https://gitlab.inria.fr/perfanalytics/pose2sim (accessed on 26 March 2022).

## Appendix A

Plots for running and cycling are provided in Appendix A.2 and Appendix A.3. Corresponding results are discussed in the main body of the article.

Although the article focused on lower limb kinematics, we ran the same analysis on sacro-lumbar, elbow, and shoulder joints (Figure A1, Figure A2, Figure A5, Figure A6, Figure A9 and Figure A10). The OpenPose model we used does not allow for the capture of wrist deviation or pronation/supination, or of any hand or finger movement.

Results were generally less good than in the lower body, especially on sacro-lumbar flexion/extension, for which all CMC values were complex. This can be attributed both to the lack of OpenPose keypoints in this area, and to the simplicity of the OpenSim model in the upper-body part. Indeed, currently all pelvis, lumbar, and thoracic angles are solely determined by the detection of the hip keypoints on the lower part, and of the shoulder and neck keypoints on the upper part. Moreover, the skeletal model did not allow for any scapulo-thoracic degree of freedom. In addition to the sacro-lumbar joint, upper-body Pearson’s correlation coefficients were mostly very good (>0.85) in most planes in walking and running. The range of motion error remained below 1° for shoulder and elbow angles in walking, while it reached almost 5° in the shoulder and 2° in the elbow in running. The mean error in the sagittal plane was below 1° in the shoulder angle in walking, but it reached 10° in the elbow; conversely, in running it reached 9° in the shoulder but remained under 1° in the elbow. In cycling, upper-body Pose2Sim angles were mostly not correlated to marker-based ones, and ROM errors and mean errors were much worse than in other tasks. Moreover, the Bland–Altman analysis showed that the data is heteroscedastic: the spread and magnitude of the errors varied as the joint angle evolved.

In conclusion, Pose2Sim does not evaluate some anatomical joint angles in the upper body, and is generally less accurate than for the lower body. This is mostly due to the lack of keypoints OpenPose detects. To date, OpenPose offers hand and face models but no detailed model of the upper limb exists. Pose2Sim could be used with other pose estimation algorithms, including custom ones leveraging DeepLabCut, for example [19], although it would involve manually labeling a large training dataset. This would enable the use of a more anatomically realistic kinematic model, such as Seth’s [55] for the shoulder girdle.
